# CHRDL1 inhibits OSCC metastasis via MAPK signaling-mediated inhibition of MED29

**DOI:** 10.1186/s10020-024-00956-y

**Published:** 2024-10-26

**Authors:** Songkai Huang, Junwei Zhang, Yu Qiao, Janak Lal Pathak, Rui Zou, ZhengGuo Piao, ShiMin Xie, Jun Liang, Kexiong Ouyang

**Affiliations:** 1 School and Hospital of Stomatology, Guangdong Engineering Research Center of Oral Restoration and Reconstruction, Guangzhou Key Laboratory of Basic and Applied Research of Oral Regenerative Medicine, Guangzhou Medical University, Guangzhou, 510182 China; 2 Department of Stomatology, The Seventh Affiliated Hospital, Sun Yat-Ssen University, Shenzhen, 518000 Guangdong China

**Keywords:** MED29, CHRDL1, OSCC, EMT

## Abstract

**Background:**

CHRDL1 belongs to a novel class of mRNA molecules. Nonetheless, the specific biological functions and underlying mechanisms of CHRDL1 in oral squamous cell carcinoma (OSCC) remain largely unexplored.

**Methods:**

RT-qPCR and immunohistochemical staining were employed to assess the mRNA and protein expression levels of the MED29 gene in clinical samples of OSCC. Additionally, RT-qPCR and Western Blot analyses were conducted to investigate the mRNA and protein expression levels of the MED29 gene specifically in OSCC. The impact of MED29 on epithelial-mesenchymal transition (EMT), invasion, and migration of OSCC was evaluated through scratch assay, transwell assay, and immunofluorescence staining. Furthermore, wound healing assay and Transwell assay were utilized to examine whether CHRDL1 influences the malignant behavior of OSCC by modulating MED29 in vitro. The regulatory role of CHRDL1 on MED29 was further elucidated in vivo through a tail vein lung metastasis model in nude mice.

**Results:**

MED29 expression was elevated in tumor tissues of OSCC patients compared with adjacent cancer tissues. Moreover, in CAL27 and SCC25 cell lines, MED29 was upregulated and associated with increased cell migration and invasion abilities. Overexpression of MED29 facilitated EMT in OSCC cell lines, whereas knockdown of MED29 impeded EMT, resulting in diminished cell migration and invasion capacities. CHRDL1 exerted inhibitory effects on the expression of MED29, thereby suppressing EMT progression and consequently restraining the invasion and migration of OSCC cells. Furthermore, CHRDL1 mediated the inhibition of migration of OSCC cell lines to the OSCC through its regulation of MED29.

**Conclusions:**

MED29 facilitated the epithelial-mesenchymal transition process in OSCC, thereby promoting migration and invasion. On the other hand, CHRDL1 exerted inhibitory effects on the invasion and metastasis of OSCC by suppressing MED29 through the inhibition of the MAPK signaling pathway.

**Supplementary Information:**

The online version contains supplementary material available at 10.1186/s10020-024-00956-y.

## Introduction

Oral squamous cell carcinoma (OSCC) represents a significant health burden, with its incidence steadily rising in recent years. Reports indicate that approximately 377,713 new cases of oral cancer are diagnosed annually, comprising about 2% of all reported malignancies worldwide. Projections suggest that this number may escalate to 510,948 cases (Yeung 2019). Despite advances in treatment, the 5-year survival rate for OSCC remains relatively low, at around 60% (Zhang et al. 2019). Consequently, the prevention, early detection, sequential therapy, and follow-up of OSCC have become paramount in the management of oral and maxillofacial tumors (Tang et al. 2021; Sung et al. 2021). Unraveling the novel biological indicators that are closely related to the occurrence and development of OSCC, can accurately and objectively evaluate the prognosis of patients with OSCC, and formulate specific personalized treatment plans to improve the survival rate and quality of life of patients with OSCC.

The invasion and metastasis of tumors are the main factors that complicate tumor treatment, and the underlying molecular mechanisms are still unclear. Epithelial-mesenchymal transition (EMT) is an important event in tumorigenesis and metastasis (Chen et al. 2019). EMT is a process in which polarized epithelial cells transform into mesenchymal cells with migration ability and acquire invasion and migration ability. EMT plays a fundamental cellular biological role in tissue repair, embryonic development, and cancer development (Jolly et al. 2019). EMT is a multi-step dynamic process. The interaction between epithelial cells disappears, the tissue structure is loose, and the cubic epithelial cells are transformed into spindle-shaped fibroblast morphology and exhibit invasiveness. The process of EMT accelerates cancer metastasis, thereby increasing cell migration and invasion, cell–matrix adhesion, intravascular infiltration and extravasation, and increasing cell viability (Bakir et al. 2020). EMT plays an important role in the process of tumor invasion and metastasis. Epithelial cells lose cell adhesion characteristics, inhibit E-cadherin expression, and increase their mobility, matrix metalloproteinase, and mesenchymal marker expression (Wang et al. 2018). At present, little is known about the EMT mechanism associated with OSCC.

Chordin-like 1 (CHRDL1) is a secreted protein that acts as an antagonist of bone morphogenetic protein (BMP) (Mulloy and Rider 2015). CHRDL1 inhibits cell proliferation and migration, and its expression is reduced in tumor tissues (Huang et al. 2020; Pei et al. 2017; Cyr-Depauw et al. 2016; Jiang et al. 2016; Li et al. 2020). Studies have confirmed that CHRDL1, as a tumor suppressor gene, inhibits the occurrence and metastasis of gastric cancer. Downregulated CHRDL1 activates Akt promoting β-catenin nuclear translocation that triggers downstream gene transcription and induces cell proliferation and EMT (Wang et al. 2020a; Chen et al. 2015). Moreover, CHRDL1 has been reported to induce migration and invasion of breast cancer cells by inhibiting the BMP signaling pathway (Deng et al. 2021; Chen et al. 2018). Our previous study unraveled that CHRDL1 expression inhibits the EMT in OSCC leading to invasion and migration by suppressing MAPK (Wu et al. 2022).

Mediator complex subunit 29 (MED29) is part of a large multiprotein coactivator complex that mediates regulatory signals from gene-specific activators to general transcription machinery in RNA polymerase II mediated transcription (Lindemann et al. 2019). The MED29 complex stimulates the assembly of the pre-initiation complex (PIC) and recruits RNA polymerase II (Pol II) to gene promoters, initiating gene expression (Vennin et al. 2019). While studies have indicated that loss of MED29 function leads to reduced mRNA levels and subsequently decreased Pol II transcriptional gene expression, its role in cancer remains unclear.

This study found a direct correlation between CHRDL1-mediated EMT and MED29 in OSCC. MED29 plays a crucial role in various biological functions in OSCC. We investigated that during the occurrence of OSCC, low expression of CHRDL1 activates the MAPK signaling pathway, which in turn activates the expression of MED29, promoting EMT in OSCC, thereby facilitating OSCC invasion and migration.

## Methods

### RT-qPCR

RNA Isolation and Quantitative Real-Time PCR Total RNA from the clinical specimens was extracted using TRIzol Reagent (Invitrogen, USA) according to the manufacturer’s instructions. Total RNA from the cells was extracted using an RNA extraction kit (19221ES50, Yeasen, China). The quantity and quality of RNAs were detected by A260/A280 with a spectrophotometer (NanoDrop 2000, Thermo Fisher Scientific, Waltham, MA, USA). Two micrograms of total RNA was used to synthesize cDNA (11141ES60, Yeasen, China). RT-qPCR was performed using a SYBR Green qPCR kit (11199ES08, Yeasen, China). Relative mRNA expression was normalized to that of the internal GAPDH control. The relative expression of targeted genes was calculated by the 2–DDCt method. Each test was repeated at least three times.

### Clinical samples

The study involving human participants obtained approval from the Ethics Committee of the School and Hospital of Stomatology, Guangzhou Medical University (KY2021026). Thirty cases of primary OSCC specimens were collected from the School and Hospital of Stomatology, Guangzhou Medical University, along with adjacent tissue samples during oral and maxillofacial surgery. Pathological diagnosis confirmed all OSCC specimens, while adjacent tissue specimens were at least 2 cm away from the resection margin and histologically diagnosed as having no precancerous lesions or reactive changes. After resection, all specimens were immediately frozen in liquid nitrogen for preservation.

### Cell line acquisition and culture

The OSCC cell lines CAL27 and SCC25 (ATCC^®^ CRL-2095™) were obtained from ATCC, while the normal oral epithelial keratinocytes cell line HOK was acquired from AULU (Guangdong, China). CAL27 and HOK cells were cultured in Dulbecco's Modified Eagle Medium (DMEM) from Gibco (Waltham, MA, USA). The complete cell culture medium included 10% fetal bovine serum (FBS) and 1% penicillin/streptomycin. For certain experiments such as wound healing assays, cell migration, and invasion assays, a serum-free cell culture medium for cancer cells was prepared using DMEM supplemented with 1% penicillin/streptomycin. All cells were maintained in a humidified incubator at 37 °C with a 5% carbon dioxide atmosphere.

### RNA sequencing

RNA sequencing was employed to identify the target genes regulated by MED29. CAL27 cells were transfected with either MED29 or NC for 48 h. Subsequently, RNA was isolated from the treated CAL27 cells using TRIzol reagent (Invitrogen, Shanghai, China). RNA-seq libraries were prepared using the KAPA Stranded RNA-Seq Library Prep Kit (Illumina, CA, USA). Sequencing was conducted by KangChen Biotech (Shanghai, China) on an Illumina HiSeq 4000 instrument. The sequencing reads were trimmed using StringTie and mapped to the human genome database (GRCh37) using Hisat2 software. Differential expression and normalized read counts (FPKM, Fragments per kilobase of gene/transcript model per million mapped fragments) were calculated using Ballgown software.

### Western blotting

Cell lysis was performed by incubating cells with ice-cold RIPA lysis buffer supplemented with a protease inhibitor cocktail (Cell Signaling Technology, Danvers, MA, USA). The lysates were centrifuged at 12,000 rpm for 10 min at 4 °C, and the supernatant was collected. Total protein was separated by electrophoresis on a 10% SDS-PAGE gel, transferred to a nitrocellulose membrane via electroblotting, and then blocked with 5% skim milk for 1 h. The membrane was then probed overnight at 4 °C with antibodies against MED29, E-cadherin, N-cadherin, and Cytokeratin 18 (Thermo Fisher, USA). Following primary antibody incubation, the membrane was incubated with goat anti-mouse or goat anti-rabbit secondary antibodies (Abcam, UK) for 2 h at room temperature. Protein bands were visualized using enhanced chemiluminescence and captured with an imaging system. The grayscale values of the bands were analyzed using Image J software.

### Transwell assay

BD Matrigel diluted in serum-free culture medium (1:8) was coated onto the upper chambers of a 24-well Transwell system and incubated at 37 °C for 2 h. CAL27\SCC25 cells were collected for each group and prepared into a cell suspension with a density of 2 × 10^5 cells/mL. Then, 100 μL of the cell suspension was added to the upper chamber, and 600 μL of DMEM containing 15% FBS was added to the lower chamber. After incubating for 24 h, cells were fixed in 4% paraformaldehyde for 20 min and stained with crystal violet solution for 15 min. Subsequently, five random fields were observed under a microscope (bright field, × 200 magnification), and cells were counted to calculate the average. To assess cell migration, all steps were repeated as described above, except that BD Matrigel was not added to the upper chamber.

### Wound healing assay

CAL27 and SCC25 cells were seeded in 6-well plates and allowed to reach 95% confluence. Using a sterile 10 μL pipette tip, cells were straight scratched, followed by washing with PBS to remove detached cells. Subsequently, cells were treated with serum-free medium containing ART (0, 10, 20, and 40 μg/mL). Images were captured in the same area at 0 and 24 h (bright field, × 100 magnification), and image analysis was performed using Image J software.

### Animal experiment

The experiment involved 4-week-old male BALB/C nude mice approved by the Ethics Committee of the Stomatology Hospital, Guangzhou Medical University (Jiangsu, China). All mouse procedures were conducted in accordance with the guidelines provided by the Animal Care Committee of the First Affiliated Hospital of Guangzhou Medical University. Mice were randomly selected from 4 different groups. Under anesthesia with 3% isoflurane, 0.1 mL of PBS containing 1 × 10^^6^ CAL27 and SCC25 suspended cells was injected into the tail vein of the mice. After being raised under standard conditions for 9 weeks, mice developed cervical dislocation and were humanely euthanized under anesthesia. OSCC tissues were then harvested, weighed, and cleaned, followed by fixation. Subsequently, the OSCC tissues were embedded in paraffin for subsequent sectioning and staining. Hematoxylin and eosin (H&E) staining was performed on the sections to assess tumor metastasis. The number of lung metastatic nodules was calculated by detailed examination of the lung tissues under a dissecting microscope.

### Small animal in vivo imaging

The experimental nude mice were intraperitoneally injected with 300 μL of 2% sodium pentobarbital (215 mg/kg) to anesthetize them. The mice were then placed in a prone position in the recording dark box of the small animal multispectral live imaging system. During the experiment, Cy7-labeled biological molecules or drugs were diluted in water and injected into the tail vein of the nude mice at a volume of 200 μL (concentration 0.5 mg/mL). One fluorescence imaging picture was taken every 5 min to analyze the distribution of fluorescent drugs within the animal’s body. During Cy7 detection, the excitation wavelength was set from 700 to 770 nm with a bandpass filter, and the emission wavelength was set at 790 nm with a longpass filter. The scanning range of the liquid crystal tunable filter was from 780 to 950 nm with a scanning step of 10 nm. The exposure time was set to 500 ms.

## Results

### MED29 was differentially expressed in OSCC patients and OSCC cell lines

**Fig. 1:**
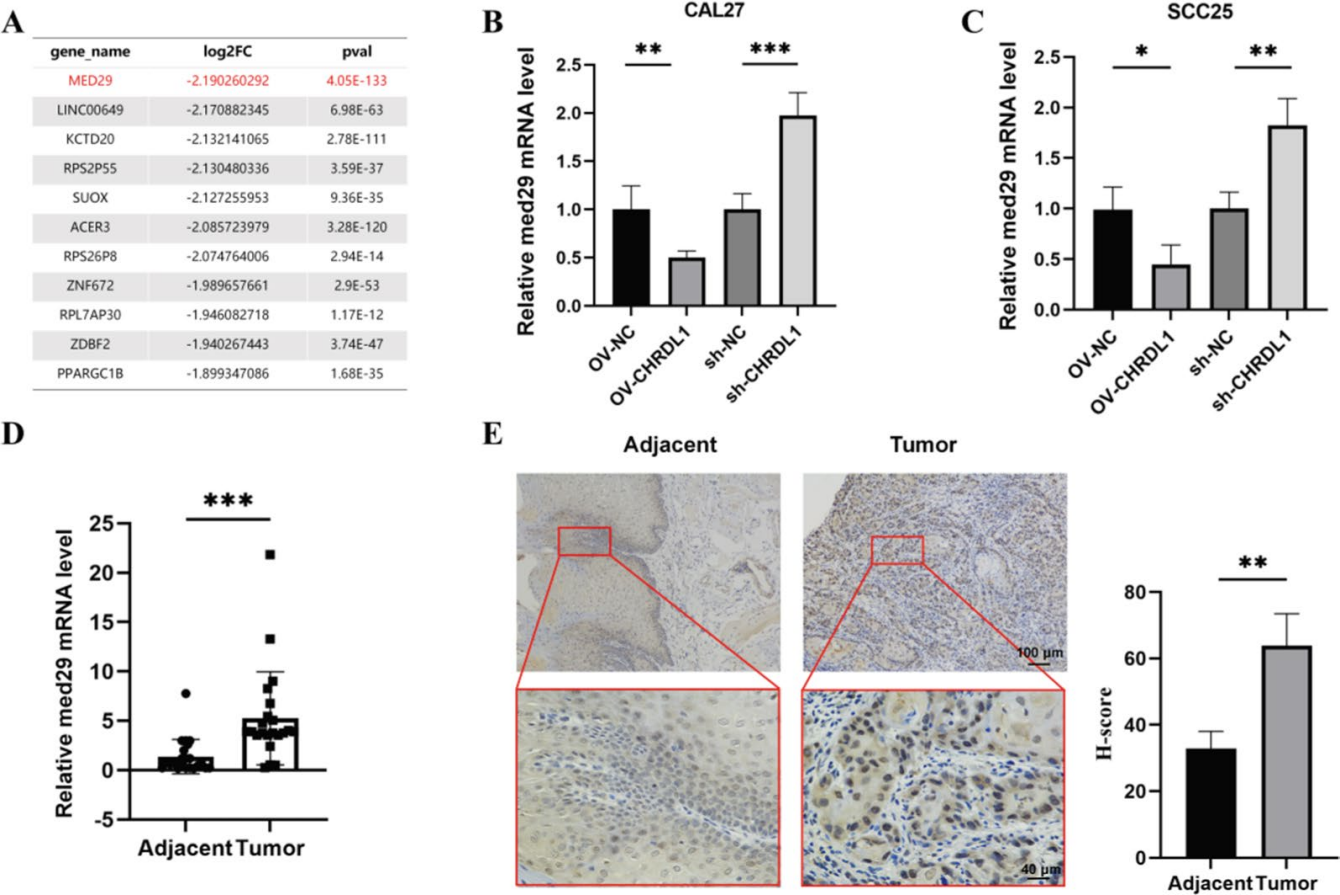
MED29 is up-regulated in OSCC and associated with worse prognosis in patients with OSCC. **A** Among the top 10 differentially expressed genes regulated by CHRDL1; **B** Validation of MED29 expression being influenced by CHRDL1 in CAL27 cell line; **C** Validation of MED29 expression being influenced by CHRDL1 in SCC25 cell line; **D** Expression of MED29 in OSCC tissue compared to adjacent non-cancerous tissue; **E** Immunohistochemical detection of MED29 expression in OSCC tissue compared to adjacent non-cancerous tissue. (*p < 0.05; **p < 0.01; ***p < 0.001)

To explore the target genes regulated by CHRDL1, we conducted an analysis using sequencing results of differentially expressed genes in oral cancer cells overexpressing CHRDL1 obtained from the NCBI database. RNA-seq analysis revealed that the transcriptome profile of cells overexpressing CHRDL1 was distinct from NC cells. Comparative analysis showed that compared to the NC control group, there were 933 upregulated genes and 879 downregulated genes in the group overexpressing CHRDL1. In the comparison between cells with CHRDL1 knockdown and the NC control group, 1037 upregulated and 828 downregulated gene expression changes were detected, respectively. Among these genes, we conducted filtering and sorting, ranking them based on the absolute value of log2FC, and selected the top 10 genes. Among the differentially expressed genes, MED29 exhibited the most significant downregulation in cells overexpressing CHRDL1 (Fig. 1a). To further understand the role of MED29 in the CHRDL1 regulatory network, we conducted lentivirus-mediated experimental manipulations of overexpression and knockdown of CHRDL1 in the CAL27 and SCC25 OSCC cell lines. We then performed RT-qPCR analysis to determine the expression levels of MED29. The results indicated that overexpression of CHRDL1 suppresses MED29 expression, while knockdown of CHRDL1 promotes MED29 expression (Fig. 1b–c). We detected the expression levels of MED29 in OSCC and adjacent non-cancerous tissues using qRT-PCR. Analysis of 30 pairs of clinical samples of OSCC revealed a significant increase in the expression of MED29 in OSCC compared to adjacent non-cancerous tissues (Fig. 1d). We utilized IHC technology to detect the expression of MED29 in selected clinical samples. The analysis revealed that in OSCC tissues, the expression of MED29 was significantly decreased compared to the control tumor tissues (Fig. 1e).

### The transcriptional and translational expression levels of MED29 in OSCC

**Fig. 2:**
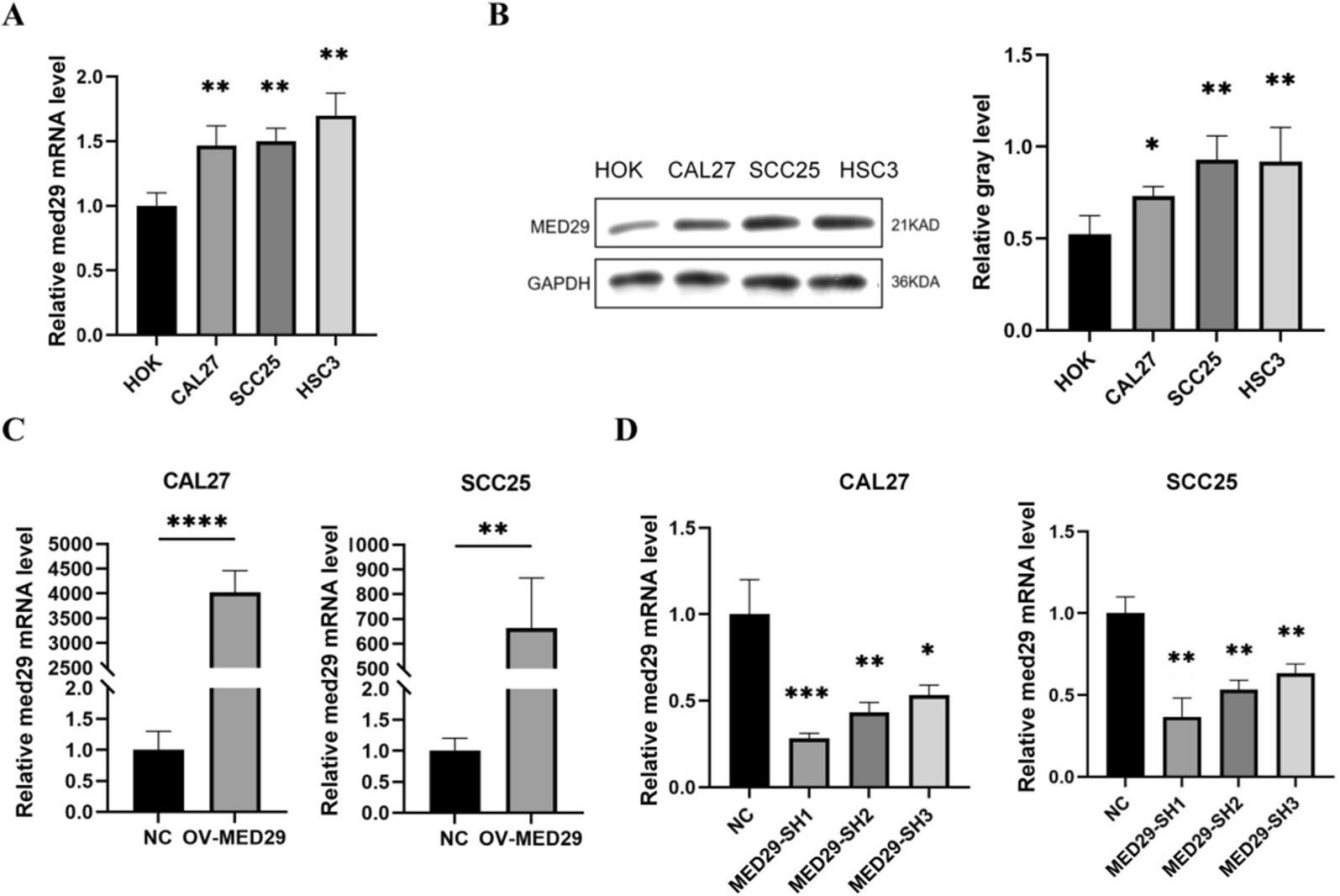
CHRDL1 enhances OSCC cell proliferation through up-regulating MED29. **A** RT-qPCR detection of mRNA expression levels of MED29 in OSCC cell lines; **B** Western blot analysis of protein expression levels of MED29 in OSCC cell lines; **C** mRNA expression levels of MED29 in CAL27 and SCC25 cells in the overexpression group; **D** mRNA expression levels of MED29 in CAL27 and SCC25 cells in the knockdown group. (*p < 0.05; **p < 0.01; ***p < 0.001; ****p < 0.0001)

To determine the transcriptional and translational levels of MED29 in OSCC, we assessed the expression of MED29 mRNA and protein using RT-qPCR and Western blot methods. We detected the mRNA and protein expression levels of MED29 in three OSCC cell lines, CAL27, SCC25, and HSC3. The experimental results indicate that MED29 is significantly upregulated in OSCC cell lines CAL27, SCC25, and HSC3 (Fig. 2a–b). To further explore the role of MED29 in the progression of OSCC, we employed lentiviral vectors to overexpress or knock down MED29 in CAL27 and SCC25 OSCC cell lines, followed by cell screening. Subsequently, we assessed the expression levels of MED29 using RT-qPCR. Experimental results indicate that in CAL27 and SCC25 cell lines, infection with lentivirus overexpressing MED29 significantly increased the levels of MED29 mRNA, confirming the successful construction of the MED29 overexpression cell models and laying the foundation for further research (Fig. 2c). Conversely, in cell lines treated with lentivirus for the knockdown, we observed a significant decrease in MED29 mRNA levels, particularly in sh1-CHRDL1-CAL27 and sh1-MED29-SCC25 cells (Fig. 2d). Based on these findings, we selected sh1-MED29-CAL27 and sh1-MED29-SCC25 cell lines as models for further experimental analysis.

### The impact of MED29 on the migration and invasion of OSCC

**Fig. 3:**
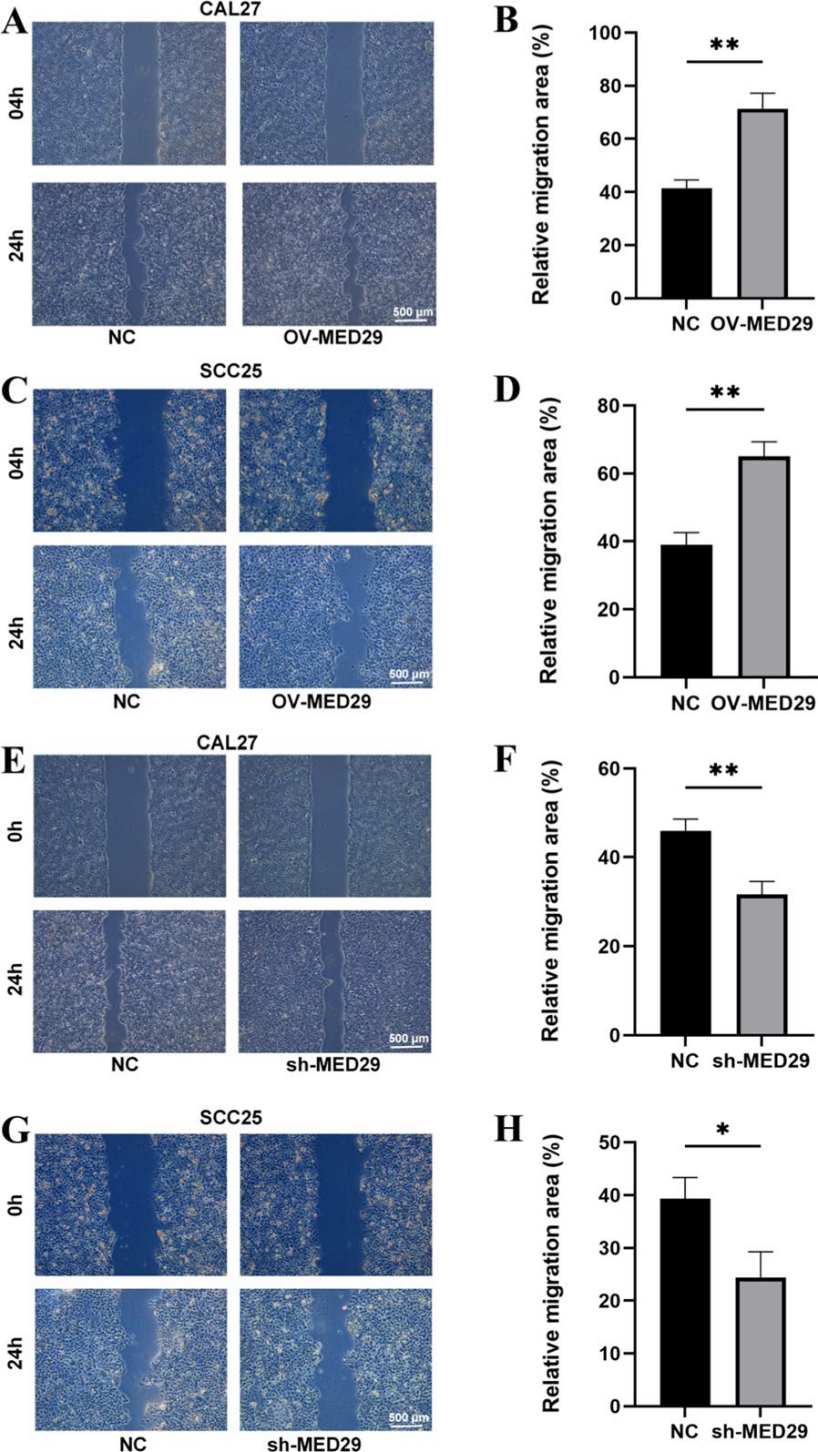
Overexpression and knockdown of MED29 inhibits CAL27/SCC25 cell migration and invasion. **A**–**D** Scratch assay of CAL27 and SCC25 cells overexpressing MED29; **E**–**H** Scratch assay and quantitative analysis results of CAL27 and SCC25 cells with MED29 knockdown (*p < 0.05; **p < 0.01)

To explore its impact on the in vitro lateral migration ability of cell lines, stable overexpression/underexpression of MED29 in OSCC cell lines was successfully established. A scratch assay was performed to study the scratch repair of MED29 OSCC cell lines for 24 h. The results of the scratch assay showed that CAL27 and SCC25 cells overexpressing MED29 exhibited significantly enhanced wound healing ability after 24 h in vitro (Fig. 3a–d). In vitro experiments demonstrated that the knockdown of MED29 had a significant inhibitory effect on wound healing ability in CAL27 and SCC25 cells (Fig. 3e–h). These findings suggest that in vitro overexpression of MED29 can promote the lateral migration ability of OSCC cell lines, while in vitro underexpression of MED29 can have an inhibitory effect.

**Fig. 4:**
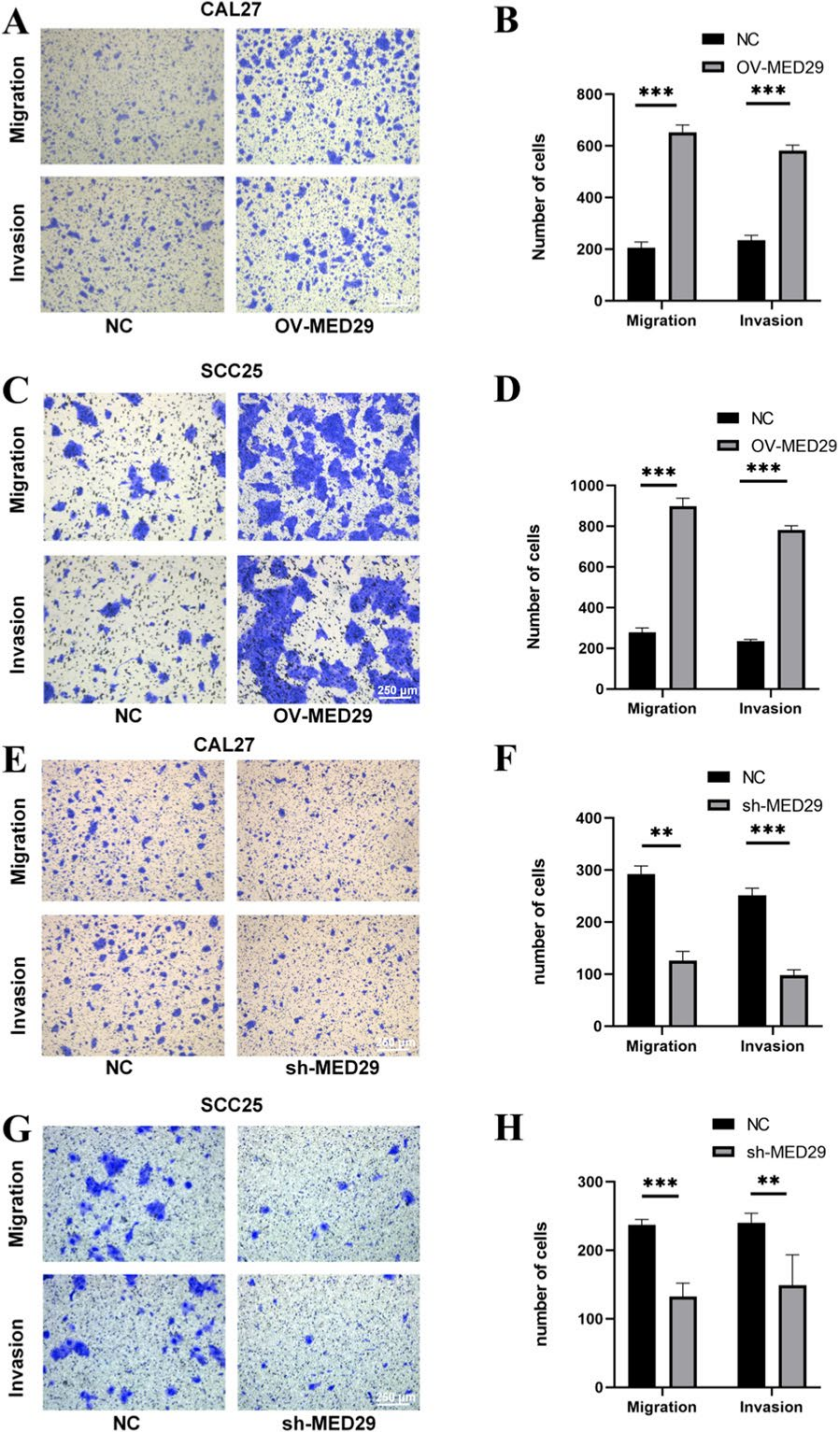
Overexpression and knockdown of MED29 inhibits CAL27/SCC25 cell migration. **A**–**D** Migration and invasion assays of CAL27 and SCC25 cells overexpressing MED29, along with quantitative analysis; **E**–**H** Migration and invasion assays of CAL27 and SCC25 cells with MED29 knockdown, along with quantitative analysis. (**p < 0.01; ***p < 0.001)

In investigating the impact of MED29 on the migration ability of OSCC cells, we conducted cell migration assays without matrix coating. We examined the effects of MED29 overexpression and knockdown on the transwell migration ability of CAL27 and SCC25 cell lines. The experimental results (Fig. 4a–d) showed that in the case of MED29 overexpression, the number of CAL27 and SCC25 cells migrating through the transwell chambers significantly increased, whereas cells with reduced MED29 expression exhibited a significant decrease in the number of migrating cells (Fig. 4e–h). This suggests that high expression of MED29 may inhibit the migration ability of these cells, while its low expression promotes the migration of OSCC cells.

### MED29 regulates EMT in OSCC cell lines

**Fig. 5:**
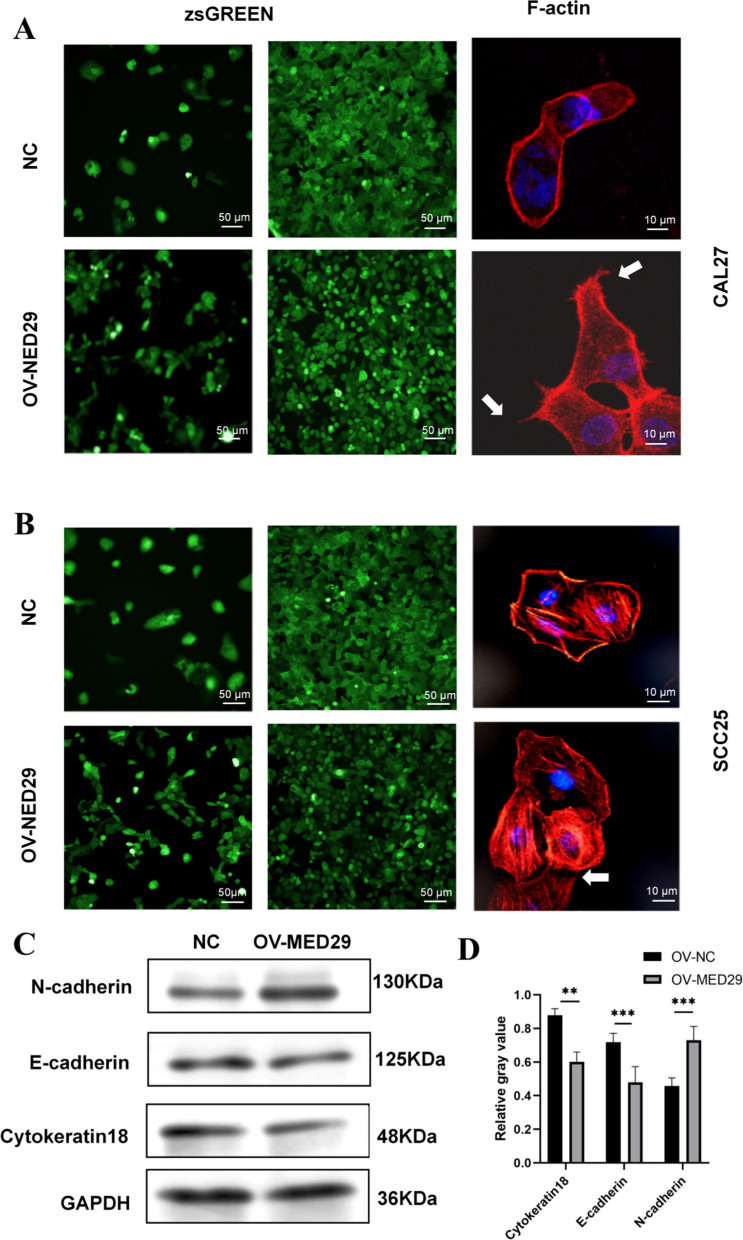
Depletion of MED29 induces the EMT phenotype. **A** In Cal27 cell line, OV-MED29 group and NC group stained with phalloidin fluorescent dye. (Left) Representative fluorescent images of cells under low cell density and (center) high cell density conditions; (right) Phalloidin fluorescent staining shows cells exhibiting filopodia, indicated by white arrows. **B** In SCC25 cell line, OV-MED29 group and NC group stained with phalloidin fluorescent dye. (Left) Representative fluorescent images of cells under low cell density and (center) high cell density conditions; (right) Phalloidin fluorescent staining shows cells exhibiting filopodia, indicated by white arrows. **C** Detection of EMT markers using Western blot method; **D** Quantitative analysis of Western blot results. (**p < 0.01; ***p < 0.001)

Overexpression of MED29 in CAL27 and SCC25 cells led to significant morphological changes. Compared to OV-NC-CAL27 and OV-NC-SCC25 cells, these changes were pronounced. OV-MED29-CAL27 and OV-MED29-SCC25 cells lost their polygonal epithelial morphology and exhibited a spindle-shaped and fibroblastic appearance (Fig. 5a–b), with abundant actin filaments, referred to as stress fibers (marked by white arrows). These findings suggest that OSCC cell lines may undergo EMT mediated by the inhibitory effect of MED29. To further validate the effect of MED29 on the EMT process in OSCC cell lines, we evaluated the expression of EMT markers at the protein level using Western blotting (Fig. 5c–d). When comparing OV-MED29 cells with control group cells, we observed several key changes. In the overexpression group, the expression levels of epithelial cell markers such as cytokeratin 18 and E-cadherin significantly increased, while the expression of the mesenchymal cell marker E-cadherin decreased significantly. Additionally, the expression levels of epithelial characteristic markers also experienced significant increases. These results suggest that high expression of MED29 promotes the EMT process in OSCC cell lines under in vitro conditions. Conversely, low expression of MED29 may inhibit this transition process. Therefore, we can conclude that MED29 plays a promoting or inhibitory role in the EMT process of OSCC cells, depending on MED29 expression level.

### CHRDL1 regulates EMT via MED29

**Fig. 6:**
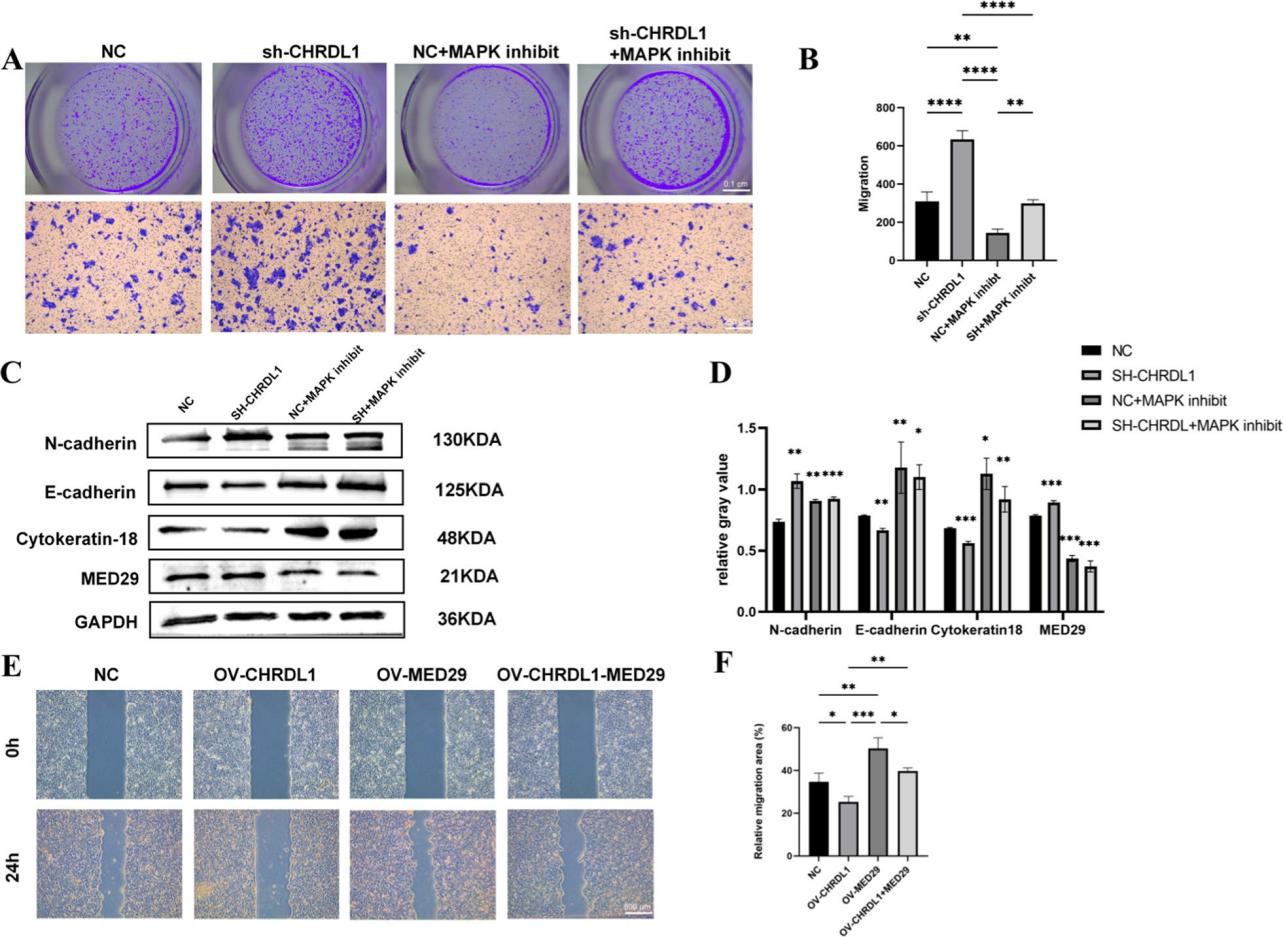
Regulation of MED29 by CHRDL1 expression. **A**–**B** MAPK inhibitor reverses the inhibitory effect of CHRDL1 knockdown on proliferation in CAL27 cells; **C–D** Western blot analysis of EMT markers expression and quantitative analysis after lentivirus transfection in CAL27 cells; **E**–**F** Scratch assay examining the reversal of CHRDL1-mediated migration inhibition by MED29. (*p < 0.05; **p < 0.01; ***p < 0.001)

When treating OSCC cells with CHRDL1 knockdown with a MAPK inhibitor, the migration rate of the knockdown group was significantly higher than that of the control group in transwell experiments, while the MAPK inhibitor reversed these defects. Compared to the control group, the addition of the MAPK inhibitor significantly reduced the migration ability in the sh1-CHRDL1-MAPK inhibit CAL27 cells (Fig. 6a–b). Thus, we further explored the downstream signaling molecules of CHRDL1 through Western Blot (Fig. 6c–d). Compared to SH-NC CAL27 cells, SH-CHRDL1 CAL27 cells showed upregulation of N-cadherin protein, cytokeratin 18, and MED29 protein, and downregulation of E-cadherin protein. Compared to sh-NC CAL27 cells, sh1-CHRDL1-MAPK inhibit CAL27 cells showed downregulation of N-cadherin protein, cytokeratin 18, and MED29 protein, and upregulation of E-cadherin protein. These results indicate that CHRDL1 may affect the MAPK pathway in OSCC cells to regulate MED29 and thus affect EMT occurrence and cell migration.

To further determine the role of MED29 in CHRDL1, a scratch assay was conducted for 24 h after co-transfecting CHRDL1 and MED29 cells, divided into four control groups. The results (Fig. 6e–f) showed that the migration ability of the CHRDL1 overexpression group was the weakest, while that of the MED29 overexpression group was the strongest. In the co-transfected group, migration was between the CHRDL1 overexpression group and the MED29 group, suggesting that MED29 can reverse the inhibitory effect of CHRDL1 on oral cancer migration, indicating that CHRDL1 plays a regulatory role in the CHRDL1-MED29 signaling axis.

### CHRDL1 affects the invasion and migration OSCC by regulating MED29

**Fig. 7:**
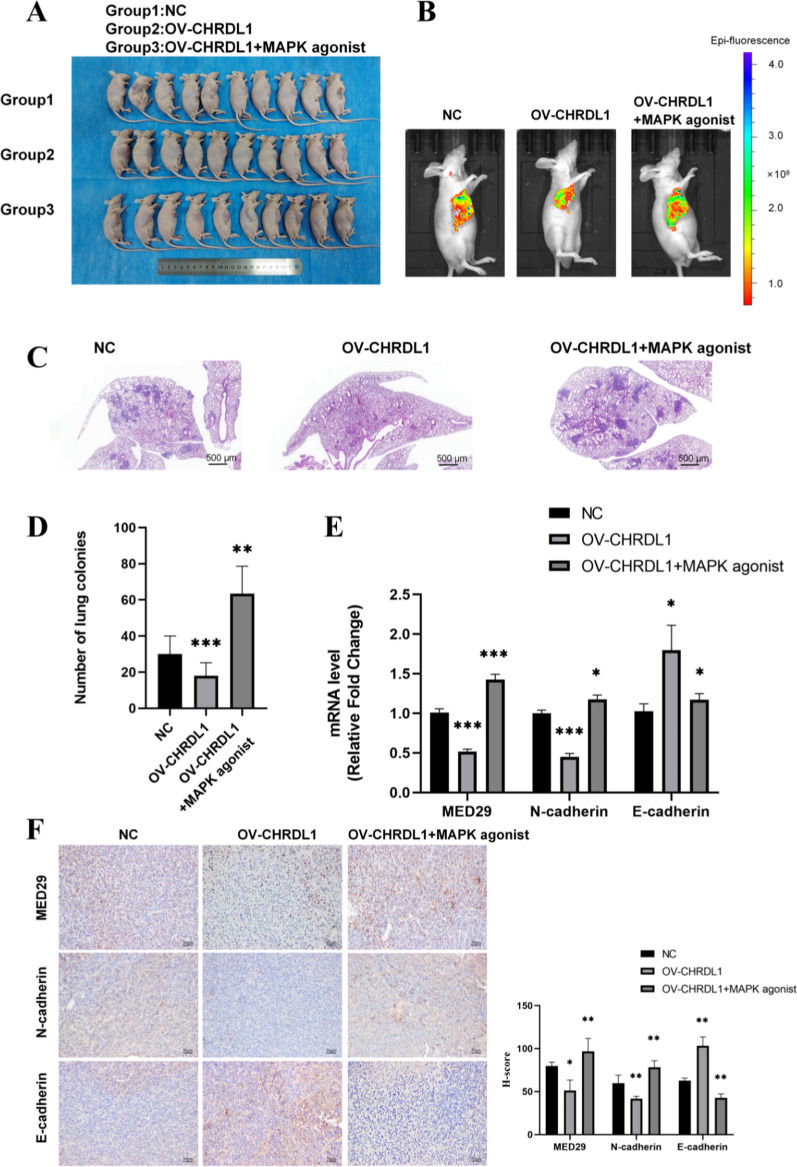
Targeting CHRDL1 Suppresses In Vivo OSCC Tumor Growth. **A** Representative images of metastatic tumors and solid tumors in nude mice; **B** Representative HE-stained histological images of xenograft tumors in nude mice; **C** In vivo imaging of nude mice; **D** Quantitative analysis of metastatic tumors; **E** Quantitative analysis of metastatic tumor weight; **F** IHC experiment detection and quantitative analysis. (*p < 0.05; **p < 0.01; ***p < 0.001)

Considering the oncogenic role of MED29 in OSCC, we hypothesized that inhibiting CHRDL1 might have therapeutic effects on OSCC. This study primarily explores the effects of CHRDL1 and MED29 on oral cancer metastasis and invasion, thus employing a tail vein injection nude mouse metastasis model. The mice were divided into three groups: control group, CHRDL1 overexpression group, and CHRDL1 overexpression + MAPK agonist group. After the experiment, the mice were photographed and analyzed (Fig. 7a). The results showed that CHRDL1 overexpression inhibited the formation of lung metastases in nude mice, while the addition of the MAPK agonist increased the number of metastatic nodules compared to the CHRDL1 overexpression group alone (Fig. 7b–d). Additionally, there was no difference in body weight among the experimental groups, while the group with the MAPK agonist showed a significant decrease in body weight (Fig. 7e). Live imaging results indicated that compared to the control group, the lung metastases in mice overexpressing CHRDL1 exhibited smaller brightness and scope (Fig. 7c). Furthermore, compared to the CHRDL1 overexpression group, the addition of the MAPK agonist resulted in significantly brighter and larger lung metastases. These results suggest that CHRDL1 may inhibit oral cancer invasion and migration through the MAPK pathway while having no effect on oral cancer proliferation.

To further explore the changes in CHRDL1-related molecules at the in vivo level, IHC experiments were conducted on tumor tissues of nude mice. The results showed that compared to the control group, overexpression of CHRDL1 inhibited the expression of MED29 and suppressed the expression of N-cadherin and p38. Moreover, after adding the MAPK agonist, the expression levels of MED29, N-cadherin, and p38 were significantly increased (Fig. 7f). This suggests that CHRDL1 suppresses OSCC invasion and migration by inhibiting the expression of MED29.

## Discussion

In this study, we analyzed the differential genes of CHRDL1 in oral cancer through NCBI sequencing data analysis, which was further validated by RT-qPCR. The experimental results suggest that CHRDL1 may exert inhibitory effects by suppressing MED29. MED29 may act as a promoting gene. Furthermore, we explored the expression levels of MED29 in OSCC and its potential regulatory functions. Additionally, we analyzed OSCC tissues and corresponding adjacent tissues using IHC methods, which also showed a significant increase in MED29 expression in OSCC tissues. This suggests that high expression of MED29 may be a factor inducing the occurrence and development of OSCC in patients. Furthermore, we validated the expression levels of MED29 at the in vitro level in CAL27, SCC25, and HSC3 cells. These findings suggest that MED29 may play a crucial role in the occurrence and development of OSCC, and its high expression may be closely associated with the biological behavior of OSCC.

There are currently few studies on MED29, especially in the field of OSCC. As a crucial component of the widely expressed Mediator complex in human embryos and adult tissues, MED29 is located in its “tail” (Sato et al. 2004;Sato et al. 2003). In fruit flies, the homologous Intersex protein of MED29 can directly interact with the DNA-binding transcription factor doublesex, collectively promoting transcriptional activity (Wang et al. 2004). In mammals, MED29 is considered a subunit of the Mediator complex responsible for transmitting signals from DNA-binding transcription factors to RNA polymerase II, thereby influencing mRNA synthesis (Kuuselo et al. 2007a; Knoll et al. 2018). Therefore, MED29 may serve as a target for one or more DNA-binding transcriptional activation factors. Studies have indicated that MED29 is a subunit gene carrying more structural chromosomal abnormalities and is overexpressed in various malignant tumors (Plaschka et al. 2015; Jeronimo and Robert 2014). However, despite its association with cancer, the mechanism of action of MED29 in the progression of OSCC remains unclear. Therefore, this study focused on the impact of MED29 on the invasion and migration of OSCC cells, aiming to elucidate its potential molecular mechanisms.

In previous studies, we observed that MED29 is overexpressed in OSCC cell lines compared to normal cells. To further investigate the function of MED29 in OSCC, this study utilized experimental methods such as scratch assays, Transwell migration, and invasion assays. By constructing OSCC cell lines with overexpression and knockdown of MED29, we further explored the role of MED29 in the occurrence and development of OSCC. MED29 promoted the invasion and migration of OSCC cells, acting as an oncogene. Conversely, inhibition of MED29 decreased OSCC cells’ migration and invasion. Studies suggest that the expression level of MED29 is closely associated with clinical pathological characteristics of tumors, such as tumor size, staging, lymph node metastasis, and distant metastasis, further demonstrating its crucial role in tumor development (Kuuselo et al. 2011, 2007b). In pancreatic cancer, MED29 regulates migration and invasion through the ERK1/2 pathway, exerting a pro-carcinogenic effect (Gu et al. 2024). In our study, CAL27 and SCC25 cells with knockdown of the MED29 gene exhibited features similar to EMT, as visually demonstrated in Figs. 2, 3 and 4. EMT is a biological process in which epithelial cells lose their typical cell adhesion characteristics and acquire the motility of mesenchymal cells, a change widely considered a critical step in cancer cell metastasis and dissemination (Dymerska and Marusiak 2024; Chatterjee et al. 2018). During EMT, the expression levels of typical cell adhesion proteins such as E-cadherin decrease, while the expression of mesenchymal cell markers promoting cell migration, such as fibronectin and N-cadherin, increases (Pei et al. 2017).

Furthermore, studies have reported that when the CHRDL1 gene is silenced, oral cancer cells also exhibit activation of EMT-like characteristics. These findings suggest that the downregulation of CHRDL1 may be a key factor triggering the EMT process in oral cancer, a process that may occur in various cancer types (Mock et al. 2015; Creyghton et al. 2010). In our experiments, when MED29 was knocked down in CAL27 and SCC25 cells, we not only observed changes in cell morphology, which resembled the changes in cell morphology during EMT, but also found corresponding changes in the expression of molecular markers at the molecular level. These results further emphasize the potential role of MED29 in regulating the EMT process of cancer cells, providing new insights into its role in tumor development and metastasis. Moreover, whether there is a correlation between CHRDL1 and MED29 warrants further investigation.

The development of OSCC is closely associated with the regulation of various molecular signaling pathways, among which the MAPK signaling pathway plays a crucial role in the tumorigenesis of OSCC (Jha et al. 2023). The MAPK signaling pathway includes key molecules such as P38, ERK1/2, and JNK, which play regulatory roles in crucial processes such as cell proliferation, differentiation, and apoptosis, exerting profound effects on the development of OSCC. Studies have shown that the activation of P38 MAPK, particularly through phosphorylation pathways, promotes the proliferation, migration, and invasion of tumor cells in OSCC, thereby exacerbating the malignancy of the tumor. Inhibiting the P38 MAPK signaling pathway not only promotes cell apoptosis and autophagy but also effectively slows down the proliferation rate of tumor cells, reduces the formation of blood vessels and lymphatic vessels, and decreases inflammation, potentially exerting inhibitory effects on OSCC progression (Vallina et al. 2021; Mock et al. 2015). Additionally, the phosphorylation of ERK1/2 also plays a crucial role in OSCC, driving cell migration, proliferation, and metastasis (Kashyap et al. 2018; Chang et al. 2021; Kim et al. 2019). This suggests that ERK1/2 may be an important regulatory factor in the proliferation and migration of OSCC cells. Regarding the JNK signaling pathway, its role in OSCC remains controversial (Chang et al. 2021). On one hand, studies have suggested that inhibition of the JNK pathway inhibits OSCC metastasis and promotes cancer cell apoptosis and autophagy (Chuang et al. 2014; Chang et al. 2012; Wang et al. 2020b). On the other hand, some studies have also indicated that downregulation of the JNK signaling pathway promotes the proliferation and migration of OSCC cells while inhibiting the intracellular autophagy process (Yang et al. 2022; Wang et al. 2004). In summary, the MAPK signaling pathway plays a key role in the development of OSCC, and the specific functions and interactions of its different molecules reveal the complexity of this signaling pathway in OSCC. In the field of OSCC, it has been found that CHRDL1 affects invasion and migration through the MAPK signaling pathway (Wu et al. 2022).

Although direct evidence may be limited, theoretically, the interaction or mutual regulation between the MAPK signaling pathway and MED29 may hold significant importance in the behavior of tumor cells. The MAPK signaling pathway, through its downstream effectors such as ERK, JNK, and P38, could potentially influence the expression or function of MED29 [51]. Studies have shown that overexpression of MED29 in COS-7 cells inhibits the transcriptional activity of SRE and AP-1, suggesting that the MED29 protein acts as a transcriptional repressor in the MAPK signaling pathway, mediating cellular functions [52]. Activation of the MAPK pathway the gene expression of MED29 through intermediary factors like transcription factors, thereby impacting the migration and invasion abilities of cells CHRDL1, acting through MED29, may affect the migration and invasion capabilities of OSCC. Aberrant activation of the MAPK pathway is often associated with malignant phenotypes in tumor cells, while overexpression or knockdown of MED29 has been shown to affect the behavior of tumor cells. Therefore, CHRDL1 may regulate MED29 through the MAPK pathway, influencing the malignant behavior of EMT in OSCC.

MED29 is involved in almost all transcriptional processes of eukaryotic cells, and its components and structures in various organ systems of the human body are specific. The importance and complexity of these changes also make the study of MED in the human body challenging. More and more studies have shown that MED29 is associated with the occurrence and development of tumors. As mentioned above, through.

In recent years, the precise treatment of tumors has made great progress, but how to make the tumor patients with insidious onset, strong invasiveness and low long-term survival rate get timely prediction and treatment is still a difficult problem to be overcome in clinical practice. At present, there is still a lack of research on the expression and biological function of MED29 in tumors. On the one hand, the mechanism of MED19 in tumor chemotherapy resistance and autophagy remains to be further explored. More new experimental methods, such as single-cell sequencing, organoid culture and multi-omics analysis, can be further applied to optimize research techniques and verify specific mechanisms. On the other hand, the specificity of the relationship between MED29 and tumor clinical stage, phenotype and prognosis needs to be further proved by more clinical trials, which is expected to be combined with big data analysis methods such as artificial intelligence, prediction model and machine learning.

**Fig. 8:**
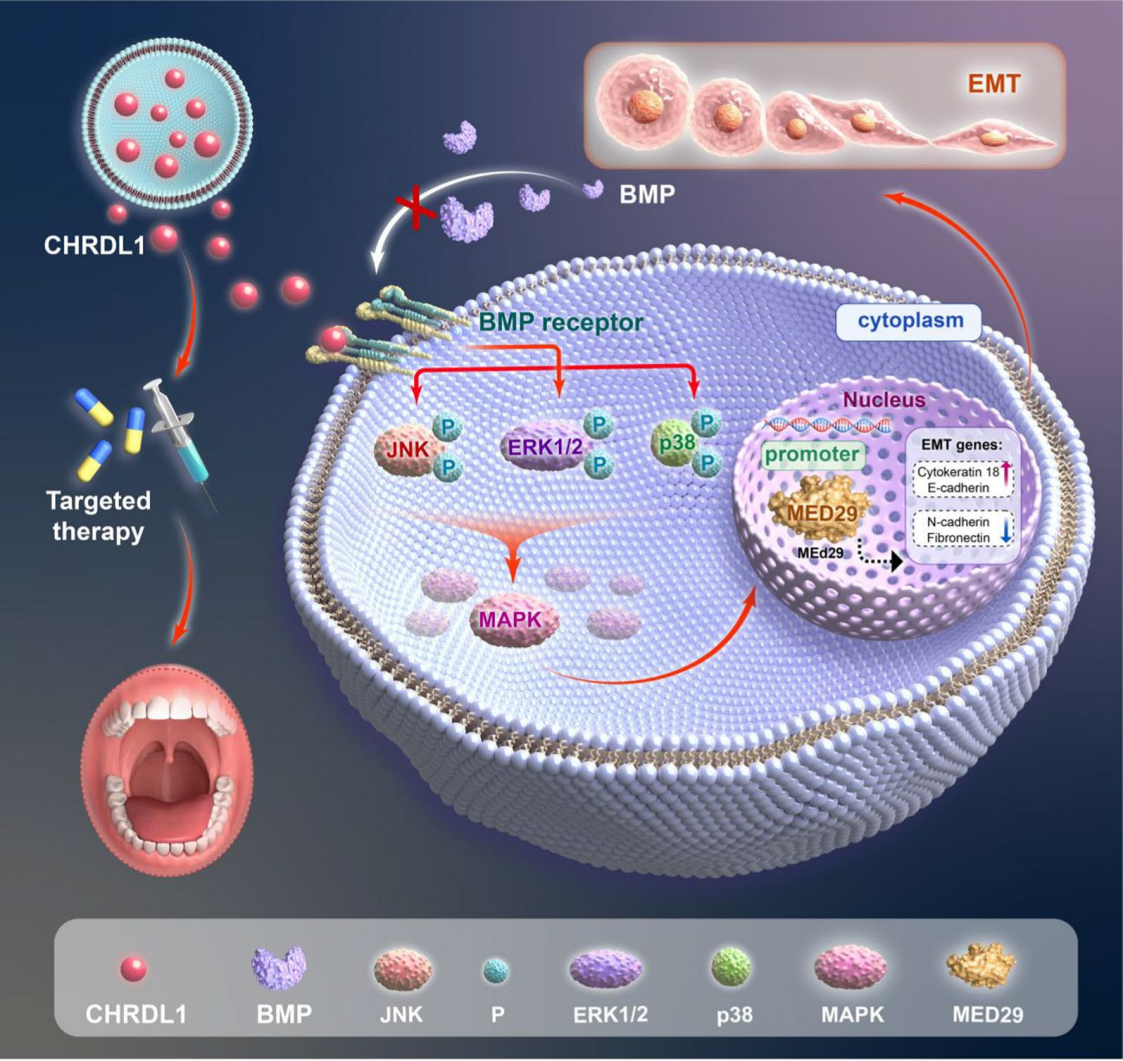
The mechanism of CHRDL1-MED29-mediated interruption of EMT evolution in OSCC. Low expression of CHRDL1 in OSCC activates the transmembrane expression of the MAPK signaling pathway, which in turn activates the MED29 promoter, promoting the overexpression of MED29. This affects the stability of the complex, ultimately facilitating the occurrence of EMT in OSCC

In summary, CHRDL1 is directly related to MED29 in the occurrence of EMT in OSCC, and the MAPK signaling pathway plays a key role in it. The low expression of CHRDL1 in OSCC activates the transmembrane expression of the MAPK signaling pathway, which in turn activates the MED29 promoter to promote the overexpression of MED29, affects the stability of the complex, and ultimately promotes the occurrence of EMT in OSCC. Therefore, the CHRDL1-MAPK pathway regulates MED29 and affects the EMT of OSCC (Fig. 8).

## Conclusions

In conclusion, we have identified CHRDL1 as a novel cancer suppressor gene in OSCC that targets the oncogenic MED29. We found that CHRDL1 can suppress the malignant behavior of OSCC cells by inhibiting the MAPK-mediated epigenetic and transcriptional regulatory axis associated with MED29. Furthermore, we confirmed that inhibition of MED29 can suppress the proliferation of OSCC cells both in vitro and in vivo. Our results underscore the significance of MED29 in CHRDL1-mediated OSCC regulation suggesting CHRDL1 and MED29 as possible diagnostic/prognostic biomarkers and novel therapeutic targets for OSCC (Fig. 8). Through further mechanism exploration in the future, MED29 may be used as. Through further mechanism exploration in the future, MED29 may be used as an effective tumor intervention target to provide new ideas and approaches for tumor treatment and prognosis in the era of precision medicine.

## Supplementary Information

Additional file 1.

## Data Availability

The datasets used and/or analyzed during the current study are available from the corresponding author upon reasonable request.
