# Supplementary material for: CHRDL1 inhibits OSCC metastasis via MAPK signaling-mediated inhibition of MED29
Source: Mol Med. 2024 Oct 26;30:187. doi: 10.1186/s10020-024-00956-y (PMC11512478; doi:10.1186/s10020-024-00956-y)
Supplement: Supplementary file 1 — Additional file 1. [file 10020_2024_956_MOESM1_ESM.pdf]

SPRINGER NATURE

Change of authorship request form - Journals (pre-acceptance)

Important information. Please read.

- This form should be used by authors to request any change in authorship (adding/deleting authors) including changes in corresponding authors. This form should not be used for name changes. Please fully complete all sections. Use black ink and block capitals and provide each author's full name with the given name first followed by the family name.

- By signing this declaration, all authors guarantee that the order of the authors are in accordance with their scientific contribution, if applicable as different conventions apply per discipline, and that only authors have been added who made a meaningful contribution to the work.

- Please note, in author collaborations where there is formal agreement for representing the collaboration, it is sufficient for the representative or legal guarantor (usually the corresponding author) to complete and sign the Authorship Change Form on behalf of all authors, **next to the added/removed author(s)**. **(Complete Section 3, followed by Section 6.)**
  In author collaborations where there is no formal agreement for representing the collaboration and **there are more than 10 authors**, one may sign for all, provided the signer appends correspondence that attests that each of the authors have agreed to the change **and the added/removed authors sign the form**. **(Complete Section 3, followed by Section 6.)**

- Please note, we cannot investigate or mediate any authorship disputes. If you are unable to obtain agreement from all authors (including those who you wish to be removed) you must refer the matter to your institution(s) for investigation. Please inform us if you need to do this.

- If you are not able to return a fully completed form within **30 days** of the date that it was sent to the author requesting the change, we may have to withdraw your manuscript. We cannot publish manuscripts where authorship has not been agreed by all authors (including those who have been removed).

- Incomplete forms will be rejected.

- Please return/upload this form, fully completed, to the Journals Editorial Office. The Journal and/or Publisher will consider the information you have provided to decide whether to approve the proposed change in authorship. We may decide to contact your institution for more information or undertake a further investigation, if appropriate, before making a final decision.

SPRINGER NATURE

Change of authorship request form - Journals (pre-acceptance)

Section 1: Please provide the current title of manuscript

Manuscript ID no.: MOME-D-24-00536R1

Title: CHRDL1 inhibits OSCC metastasis via MAPK signaling-mediated inhibition of MED29

Section 2: Please provide the previous authorship, in the order shown on the manuscript before the changes were introduced. Please indicate the corresponding author by adding (CA) behind the name.

	First name(s)	Family name	ORCID or SCOPUS id, if available
1st author	Songkai	Huang	
2nd author	Junwei	Zhang (co-first authors)	
3rd author	Yu	Qiao	
4th author	Janak	Pathak	
5th author	Rui	Zhou	
6th author	ZhengGuo	Piao	
7th author	ShiMin	Xie	
8th author	Jun	Liang (Corresponding authors)	
9th author	Kexiong	Ouyang (Corresponding authors)	
10th author			

Please use an additional sheet if there are more than 10 authors.

SPRINGER NATURE Springer Nature is one of the world's leading global research, educational and professional publishers, created in May 2015 through the combination of Nature Publishing Group, Palgrave Macmillan, Macmillan Education and Springer Science+Business Media.

SPRINGER NATURE

Change of authorship request form - Journals (pre-acceptance)

Section 3: Please provide a justification for change. Please use this section to explain your reasons for changing the authorship of your manuscript, e.g. what necessitated the change in authorship? Please refer to the (journal) policy pages for more information about authorship. Please explain why omitted authors were not originally included and/or why authors were removed on the submitted manuscript.

After further review and discussion, we found that some contributors were not properly recognized initially, while others were mistakenly listed as authors.Omitted Authors: The omitted authors made significant contributions in the early stages of the research, including data analysis, critical revisions, and intellectual input. These contributions were not fully recognized at the time of the initial submission.We have reassessed all contributions, and the current authorship accurately reflects those who made significant contributions to the conception, design, execution, and interpretation of the study.

Section 4: Proposed new authorship. Please provide your new authorship list in the order you would like it to appear on the manuscript. Please indicate the corresponding author by adding (CA) behind the name. <u>If the Corresponding Author has changed, please indicate the reason under section 3</u>.

	First name(s)	Family name (this name will appear in full on the final publication and will be searchable in various abstract and indexing databases)	Affiliated institute	E-mail address
1st author	Songkai	Huang	Stomatology Hospital of Guangzhou Medical University	15625849554@163.com
2nd author	Junwei	Zhang	Stomatology Hospital of Guangzhou Medical University	dearcanon123@126.com
3rd author	Yu	Qiao	The Seventh Affiliated Hospital, Sun Yat-Sen University	pathak@gzhmu.edu.cn
4th author	Janak	Pathak	Stomatology Hospital of Guangzhou Medical University	loryen@126.com
5th author	Rui	Zhou	Stomatology Hospital of Guangzhou Medical University	zourui2009@hotmail.com
6th author	ZhengGuo	Piao	Stomatology Hospital of Guangzhou Medical University	zhengguopiao@hotmail.com
7th author	ShiMin	Xie	Stomatology Hospital of Guangzhou Medical University	986390741@qq.com
8th author	Jun	Liang (CA)	The Seventh Affiliated Hospital, Sun Yat-Sen University	liangjun@mail.sysu.edu.cn
9th author	Kexiong	Ouyang (CA)	Stomatology Hospital of Guangzhou Medical University	ouyangkexiong@163.com
10th author				

Please use an additional sheet if there are more than 10 authors.

SPRINGER NATURE Springer Nature is one of the world’s leading global research, educational and professional publishers, created in May 2015 through the combination of Nature Publishing Group, Palgrave Macmillan, Macmillan Education and Springer Science+Business Media.

Change of authorship request form - Journals (pre-acceptance)

Section 5: Author contribution, Acknowledgement and Disclosures. Please use this section to provide a new disclosure statement and, if appropriate, acknowledge any contributors who have been removed as authors and ensure you state what contribution any new authors made (if applicable per the journal or book (series) policy). Please ensure these are updated in your manuscript - after approval of the change(s) - as our production department will not transfer the information in this form to your manuscript.

New acknowledgements:

We would like to acknowledge the contributions of Janak Lal Pathak, who provided valuable administrative and technical support during the initial phases of the research.

New Disclosures (financial and non-financial interests, funding):

The authors declare no conflicts of interest related to this study. All contributors who were removed from the author list have been acknowledged for their contributions, and the inclusion of new authors reflects their significant contributions to the study.

New Author Contributions statement (if applicable per the journal policy):

SH and JZ contributed to the conception and design of the study. SH, JZ, YQ, and RZ performed the experiments. SH, JZ, JLP,YQ, RZ, ZP,, SX, and KOY performed the statistical analysis. QW wrote the first draft of the manuscript. SH, JZ, JL, and KOY critically revised the manuscript. All authors contributed to the article and approved the submitted version.

State 'Not applicable' if there are no new authors.

SPRINGER NATURE Springer Nature is one of the world's leading global research, educational and professional publishers, created in May 2015 through the combination of Nature Publishing Group, Palgrave Macmillan, Macmillan Education and Springer Science+Business Media.

SPRINGER NATURE

Change of authorship request form - Journals (pre-acceptance)

Section 6: Declaration of agreement. *All* authors, unchanged, new and removed *must* sign this declaration.
(NB: Please print the form, (docu)-sign and return/upload a scanned copy. Please note that signatures that have been inserted as an image file are acceptable as long as it is handwritten. Typed names in the signature box are unacceptable.) *** Please delete as appropriate. Delete all of the bold if you were on the original authorship list and are remaining as an author.**

	First name	Family name		Signature	Date
1st author	Songkai	Huang	I agree to the proposed new authorship shown in section 4 **/and the addition/removal*of my name to the authorship list** /and the proposed change in corresponding author	SongKai Huang	12/7/2024
2nd author	Junwei	Zhang	I agree to the proposed new authorship shown in section 4 **/and the addition/removal*of my name to the authorship list** /and the proposed change in corresponding author	Junwei zhang	12//7/2024
3rd author	Yu	Qiao	I agree to the proposed new authorship shown in section 4 **/and the addition/removal*of my name to the authorship list** /and the proposed change in corresponding author	Yu Qiao	13/7/2024
4th authors	Janak	Pathak	I agree to the proposed new authorship shown in section 4 **/and the addition/removal*of my name to the authorship list** /and the proposed change in corresponding author	Janak	13/7/2024
5th author	Rui	Zhou	I agree to the proposed new authorship shown in section 4 **/and the addition/removal*of my name to the authorship list** /and the proposed change in corresponding author	Rui zhou	12//7/2024
6th author	ZhengGuo	Piao	I agree to the proposed new authorship shown in section 4 **/and the addition/removal*of my name to the authorship list** /and the proposed change in corresponding author	zhengGuo Piao	12//7/2024
7th author	ShiMin	Xie	I agree to the proposed new authorship shown in section 4 **/and the addition/removal*of my name to the authorship list** /and the proposed change in corresponding author	Shimin Xie	12//7/2024

SPRINGER NATURE Springer Nature is one of the world's leading global research, educational and professional publishers, created in May 2015 through the combination of Nature Publishing Group, Palgrave Macmillan, Macmillan Education and Springer Science+Business Media.

	First name	Family name		Signature	Date
8th author	Jun	Liang	I agree to the proposed new authorship shown in section 4 /**and the addition/removal*of my name to the authorship list** /and the proposed change in corresponding author	Jun Liang	12//7/2024
9th author	Kexiong	Ouyang	I agree to the proposed new authorship shown in section 4 /**and the addition/removal*of my name to the authorship list** /and the proposed change in corresponding author	kexiong Ouyang	12//7/2024
10th author			I agree to the proposed new authorship shown in section 4 /**and the addition/removal*of my name to the authorship list** /and the proposed change in corresponding author		

Please use an additional sheet if there are more than 10 authors.

In case of author collaborations with formal agreement:

	Name of consortium/consortia	First name	Family name		Signature	Date
Representative/legal guarantor				I agree to the proposed new authorship shown in section 4 /**and the addition/removal*of my name to the authorship list** /and the proposed change in corresponding author		

Both added/removed authors should complete the information in the first table under Section 6.

---- End of form ----

SPRINGER NATURE Springer Nature is one of the world's leading global research, educational and professional publishers, created in May 2015 through the combination of Nature Publishing Group, Palgrave Macmillan, Macmillan Education and Springer Science+Business Media.
